# Supplementary material for: Role of Health Professionals Regarding the Impact of Climate Change on Health—An Exploratory Review
Source: Int J Environ Res Public Health. 2021 Mar 20;18(6):3222. doi: 10.3390/ijerph18063222 (PMC8003659; doi:10.3390/ijerph18063222)
Supplement: Supplementary file 1 [file ijerph-18-03222-s001.pdf]

**Supplementary 1. PubMed search strategy**

("Climate Change"[Mesh] OR "Environment"[MeSH] OR "Environmental Health"[Mesh:NoExp] OR "Heat Stress Disorders"[MeSH] OR climat*[tiab] OR "conservation of natural resources"[tiab] OR ecosystem[tiab] OR (environment*[tiab] AND impact*[tiab] AND health[tiab]) OR "extreme heat"[tiab] OR "global warming"[tiab] OR greenhouse effect*[tiab] OR "heat stress"[tiab] OR "sustainable development"[tiab]) AND ("Health Personnel"[MeSH] OR "health personnel"[tiab] OR healthcare provider*[tiab] OR nurse*[tw] OR physician*[tw]) AND ("Role"[Mesh] OR "Communication"[Mesh:NoExp] OR "Implementation Science"[Mesh] OR "Information Literacy"[Mesh] OR "Information Dissemination"[Mesh] OR "Education, Professional"[Mesh] OR "Health Education"[Mesh] OR "consumer health information"[tiab] OR "information dissemination"[tiab] OR "information literacy"[tiab] OR "nurse's role"[tiab] OR "physician's role"[tiab]) NOT ("Animals"[MeSH] NOT "Humans"[MeSH]) AND (("2000/01/01"[PDAT] : "2020/12/31"[PDAT]))

**Supplementary 2. Full list of articles included in the review**


1. Adlong W, Dietsch E. Nursing and climate change: An emerging connection. Collegian. 2015;22(1):19–24.
2. Agarwal BB. Journey of the carbon-literate and climate-conscious endosurgeon having a head, heart, hands, and holistic sense of responsibility. Surg Endosc. 2008 Dec;22(12):2539–40.
3. Alame D, Truog RD. How Should Clinicians Weigh the Benefits and Harms of Discussing Politicized Topics that Influence Their Individual Patients' Health? AMA J Ethics. 2017 Dec 1;19(12):1174–82.
4. Allen CE. The challenges of climate change. Ala Nurse. 2009 Feb;36(4):11–6; quiz 17.
5. Armstrong F. Do no harm? Health care and the environment. Aust Nurs J. 2005 Feb;12(7):18–21.
6. Auerbach PS. Physicians and the environment. JAMA. 2008 Feb 27;299(8):956–8.
7. Barlow G. Nurses feel impact of climate change. Aust Nurs J. 2008 May;15(10):24–6.
8. Barna S, Goodman B, Mortimer F. The health effects of climate change: what does a nurse need to know? Nurse Educ Today. 2012 Oct;32(7):765–71.
9. Bedsworth L. Preparing for climate change: a perspective from local public health officers in California. Environ Health Perspect. 2009 Apr;117(4):617–23.
10. Belkin G. Leadership for the Social Climate. N Engl J Med. 2020 May 21;382(21):1975–7.
11. Bell E. Readying health services for climate change: a policy framework for regional development. Am J Public Health. 2011 May;101(5):804–13.
12. Bell EJ. Climate change: what competencies and which medical education and training approaches? BMC Med Educ. 2010 Apr 30;10:31.
13. Bhutta ZA, Aimone A, Akhtar S. Climate change and global child health: what can paediatricians do? Arch Dis Child. 2019 May;104(5):417–8.
14. Blakemore S. Nurses urged to take a lead in reducing the NHS carbon footprint. Nurs Stand. 2009 Feb 4;23(22):12–3.
15. Brucker MC. Nursing the Planet. Nurs Womens Health. 2019 Dec;23(6):461–2.
16. Burkle FM. Pandering to ignorance on climate change: lessons from an investment strategist. Prehosp Disaster Med. 2013 Jun;28(3):200–1.
17. Butler CD. Lightening our carbon footprint: economics, norms and doctors. Med J Aust. 2010

May 3;192(9):485–6.
18. Butler CD, Harley D. Primary, secondary and tertiary effects of eco-climatic change: the medical response. Postgrad Med J. 2010 Apr;86(1014):230–4.
19. Capon AG, Talley Ac NJ, Horton RC. Planetary health: what is it and what should doctors do? Med J Aust. 2018 Apr 16;208(7):296–7.
20. Carr JL, Sheffield PE, Kinney PL. Preparedness for climate change among local health department officials in New York state: a comparison with national survey results. J Public Health Manag Pract. 2012 Apr;18(2):E24-32.
21. Chalupka S, Anderko L. Climate Change and Schools: Implications for Children's Health and Safety. Creat Nurs. 2019 Aug 15;25(3):249–57.
22. Chenven L, Copeland D. Front-line worker engagement: greening health care, improving worker and patient health, and building better jobs. New Solut. 2013 Jan 1;23(2):327–45.
23. Chivian E. Why doctors and their organisations must help tackle climate change: an essay by Eric Chivian. BMJ. 2014 Apr 2;348:g2407.
24. Cook C, Demorest SL, Schenk E. Nurses and Climate Action. Am J Nurs. 2019 Apr;119(4):54–60.
25. Costello A, Montgomery H, Watts N. Climate change: the challenge for healthcare professionals. BMJ. 2013 Oct 9;347:f6060.
26. Couser G. Emergency medicine and environmental activism. Emerg Med Australas. 2004 Dec;16(5–6):469–72.
27. Cowell JM. Climate Change, School Health and School Nursing: A Call to Action. J Sch Nurs. 2019 Dec;35(6):394.
28. Crowley RA, Health and Public Policy Committee of the American College of Physicians. Climate Change and Health: A Position Paper of the American College of Physicians. Ann Intern Med. 2016 May 3;164(9):608–10.
29. Cruz JP, Felicilda-Reynaldo RFD, Alshammari F, Alquwez N, Alicante JG, Obaid KB, et al. Factors Influencing Arab Nursing Students' Attitudes toward Climate Change and Environmental Sustainability and their Inclusion in Nursing Curricula. Public Health Nurs. 2018 Nov;35(6):598–605.
30. Demorest S, Spengeman S, Schenk E, Cook C, Weston HL. The Nurses Climate Challenge: A National Campaign to Engage 5,000 Health Professionals Around Climate Change. Creat Nurs. 2019 Aug 15;25(3):208–15.
31. Douglas K. Nurses feel the heat. Aust Nurs Midwifery J. 2014 Feb;21(7):20–4.
32. Dunk JH, Jones DS. Sounding the Alarm on Climate Change, 1989 and 2019. N Engl J Med. 2020 Jan 16;382(3):205–7.
33. Eide P, Odom-Maryon T. Environmental and Climate Change Initiatives in Nursing Education. Annu Rev Nurs Res. 2019 Dec 23;38(1):131–44.
34. Every-Palmer S, McBride S, Berry H, Menkes DB. Climate change and psychiatry. Aust N Z J Psychiatry. 2016 Jan;50(1):16–8.
35. Friedrich MJ. Medical Community Gathers Steam to Tackle Climate's Health Effects. JAMA. 2017 Apr 18;317(15):1511–3.
36. George M, Bruzzese J-M, Matura LA. Climate Change Effects on Respiratory Health: Implications for Nursing. J Nurs Scholarsh. 2017 Nov;49(6):644–52.

37. Gill M, Godlee F, Horton R, Stott R. Doctors and climate change. BMJ. 2007 Dec 1;335(7630):1104–5.
38. Gill M, Stott R. Health professionals must act to tackle climate change. Lancet. 2009 Dec 12;374(9706):1953–5.
39. Gómez A, Balsari S, Nusbaum J, Heerboth A, Lemery J. Perspective: Environment, biodiversity, and the education of the physician of the future. Acad Med. 2013 Feb;88(2):168–72.
40. Goodman B. The need for a "sustainability curriculum" in nurse education. Nurse Educ Today. 2011 Nov;31(8):733–7.
41. Gould RM. The physician's role in efforts to slow global warming. Am Fam Physician. 2011 Aug 1;84(3):256–7.
42. Grabow M, Bryan T, Checovich M, Converse A, Middlecamp C, Mooney M, et al. Mindfulness and Climate Change Action: A Feasibility Study. Sustainability. 2018 May;10(5).
43. Green EIH, Blashki G, Berry HL, Harley D, Horton G, Hall G. Preparing Australian medical students for climate change. Aust Fam Physician. 2009 Sep;38(9):726–9.
44. Guggenheim R. The health threat of climate change: working in partnership with patients. Br J Gen Pract. 2016 Mar;66(644):149.
45. Guggenheim R. Bringing the planet into the generalist practice: a form of preventive care. Lancet Planet Health. 2018 May;2(5):e200–1.
46. Haines A, Ebi K. The Imperative for Climate Action to Protect Health. N Engl J Med. 2019 Jan 17;380(3):263–73.
47. Haines A, Scheelbeek P. The health case for urgent action on climate change. BMJ. 2020 Mar 30;368:m1103.
48. Hale I, Hale D, Howard C, Bell W. Time to divest from the fossil-fuel industry. CMAJ. 2014 Sep 2;186(12):960.
49. Herman B. Health systems urged to divest fossil-fuel stocks as UK doctors act. Mod Healthc. 2014 Aug 4;44(31):8–9.
50. Horton G, Magin P. Healthy patients, healthy planet--green recommendations for GP health promotion. Aust Fam Physician. 2007 Dec;36(12):1006–8.
51. Isaacs D. Climate change: The moral role of paediatricians. J Paediatr Child Health. 2015 Sep;51(9):843–4.
52. Jay M, Marmot MG. Health and climate change. Lancet. 2009 Sep 19;374(9694):961–2.
53. Kadandale S, Marten R, Dalglish SL, Rajan D, Hipgrave DB. Primary health care and the climate crisis. Bull World Health Organ. 2020 Nov 1;98(11):818–20.
54. Kefford RF. Medical heat for climate change. Med J Aust. 2006 Jun 5;184(11):582.
55. Kemper KJ, Etzel RA. Research about climate advocacy: Directions from a pilot survey of academic pediatricians. Complement Ther Med. 2020 Mar;49:102335.
56. Kerr R, Nerbonne JF, Potter T. Sparking a Movement for a Healthy Climate Through Leadership Development. Creat Nurs. 2019 Aug 15;25(3):216–21.
57. Khalifian S, Rosenbach M. Dermatology, climate change, and the perils of attacks on expertise. J Am Acad Dermatol. 2018 Aug;79(2):397–9.
58. Kiang K, Graham S, Farrant B. Climate change, child health and the role of the paediatric profession in under-resourced settings. Trop Med Int Health. 2013 Sep;18(9):1053–6.

59. Kirk M. The impact of globalization and environmental change on health: challenges for nurse education. Nurse Educ Today. 2002 Jan;22(1):60–71; discussion 72-75.
60. Kitt-Lewis E, Adam M, Buckland P, Clark D, Hockenberry K, Jankura D, et al. Creating a Generation of Sustainable Nurses: Sustainability Efforts in Nursing Education. Nurs Clin North Am. 2020 Mar;55(1):1–10.
61. Kurth AE. Planetary Health and the Role of Nursing: A Call to Action. J Nurs Scholarsh. 2017 Nov;49(6):598–605.
62. Ladouceur R. Our fight against climate change. Can Fam Physician. 2019 Nov;65(11):766.
63. Laking G, Woodward A, Metcalfe S, Macmillan A, Lindsay G, Santa Barbara J, et al. Climate science, denial and the Declaration of Delhi. N Z Med J. 2009 Dec 11;122(1307):84–93.
64. Laustsen G. Greening in healthcare. Nurs Manage. 2010 Nov;41(11):26–31.
65. Leffers J, Butterfield P. Nurses play essential roles in reducing health problems due to climate change. Nurs Outlook. 2018 Apr;66(2):210–3.
66. Leffers J, Levy RM, Nicholas PK, Sweeney CF. Mandate for the Nursing Profession to Address Climate Change Through Nursing Education. J Nurs Scholarsh. 2017 Nov;49(6):679–87.
67. Lopez-Medina IM, Álvarez-Nieto C, Grose J, Elsbernd A, Huss N, Huynen M, et al. Competencies on environmental health and pedagogical approaches in the nursing curriculum: A systematic review of the literature. Nurse Educ Pract. 2019 May;37:1–8.
68. MacPherson CC. Time for physicians to take action on climate change. Acad Med. 2009 Jul;84(7):817.
69. Macpherson CC, Wynia M. Should Health Professionals Speak Up to Reduce the Health Risks of Climate Change? AMA J Ethics. 2017 Dec 1;19(12):1202–10.
70. Madden DL, McLean M, Horton GL. Preparing medical graduates for the health effects of climate change: an Australasian collaboration. Med J Aust. 2018 Apr 16;208(7):291–2.
71. Maibach EW, Chadwick A, McBride D, Chuk M, Ebi KL, Balbus J. Climate change and local public health in the United States: preparedness, programs and perceptions of local public health department directors. PLoS One. 2008 Jul 30;3(7):e2838.
72. Maibach EW, Kreslake JM, Roser-Renouf C, Rosenthal S, Feinberg G, Leiserowitz AA. Do Americans Understand That Global Warming Is Harmful to Human Health? Evidence From a National Survey. Ann Glob Health. 2015 Jun;81(3):396–409.
73. Maibach EW, Sarfaty M, Mitchell M, Gould R. Limiting global warming to 1.5 to 2.0°C-A unique and necessary role for health professionals. PLoS Med. 2019 May;16(5):e1002804.
74. McCarthy G, Bernstein A. Combating EPA Rollbacks - Health Care's Response to a Retreat on Climate. N Engl J Med. 2019 Aug 22;381(8):696–8.
75. McCartney G, Hanlon P. What can health professionals contribute to the challenge of sustainability? Public Health. 2009 Dec;123(12):761–4.
76. McCartney PR. Climate change and child health. MCN Am J Matern Child Nurs. 2007 Aug;32(4):255.
77. McDermott RA. The carbon footprints of obesity, chronic disease and population growth: four things doctors can do. Med J Aust. 2010 May 3;192(9):531–2.
78. McDermott-Levy R, Jackman-Murphy KP, Leffers JM, Jordan L. Integrating Climate Change Into Nursing Curricula. Nurse Educ. 2019 Feb;44(1):43–7.

79. Mehta A. Physicians' contribution to climate change. CMAJ. 2009 May 26;180(11):1176.
80. Melamed A, Jackson N. Nursing and environmental health. Health Prog. 2003 Dec;84(6):29–32, 53.
81. Mendes A. Greener treatments: the NHS carbon footprint. Br J Community Nurs. 2019 May 2;24(5):248–9.
82. Metcalfe S, Woodward A, Macmillan A, Baker M, Howden-Chapman P, Lindsay G, et al. Why New Zealand must rapidly halve its greenhouse gas emissions. N Z Med J. 2009 Oct 9;122(1304):72–95.
83. Morgan RE. Determined Action to Tackle Health Determinants: A Collaborative Response to the Challenge of Climate Change Mitigation in Practice Settings. Creat Nurs. 2019 Aug 15;25(3):195–200.
84. Morrison L. Viewpoint: Doctors and Extinction Rebellion. Br J Gen Pract. 2019 Jul;69(684):344.
85. Neal-Boylan L, Breakey S, Nicholas PK. Integrating Climate Change Topics Into Nursing Curricula. J Nurs Educ. 2019 Jun 1;58(6):364–8.
86. Nicholas PK, Breakey S. Climate Change, Climate Justice, and Environmental Health: Implications for the Nursing Profession. J Nurs Scholarsh. 2017 Nov;49(6):606–16.
87. O'Day T, Wallace C. Members take action to reduce effects of climate change. Am Nurse. 2015 Oct;47(5):13.
88. Parker CL. Slowing global warming: benefits for patients and the planet. Am Fam Physician. 2011 Aug 1;84(3):271–8.
89. Parker CL, Wellbery CE, Mueller M. The Changing Climate: Managing Health Impacts. Am Fam Physician. 2019 Nov 15;100(10):618–26.
90. Patil M, Jeffery KJ. What does climate change mean for occupational health professionals? Occup Med (Lond). 2020 Sep 9;70(6):386–8.
91. Patrick K. Physicians and climate change policy: we are powerful agents of change. CMAJ. 2015 Mar 17;187(5):307.
92. Patrick R, Capetola T. It's here! Are we ready? Five case studies of health promotion practices that address climate change from within Victorian health care settings. Health Promot J Austr. 2011 Dec;22 Spec No:S61-67.
93. Phipps R, Macmillan A, Metcalfe S, Mansoor O, Lindsay G, Metcalfe S, et al. Sign On--prescribing for climate health. N Z Med J. 2009 Oct 30;122(1305):106–8.
94. Phipps R, Randerson R, Blashki G. The climate change challenge for general practice in New Zealand. N Z Med J. 2011 Apr 29;124(1333):47–54.
95. Potter T. Planetary Health: The Next Frontier in Nursing Education. Creat Nurs. 2019 Aug 15;25(3):201–7.
96. Rabinowitz PM, Natterson-Horowitz BJ, Kahn LH, Kock R, Pappaioanou M. Incorporating one health into medical education. BMC Med Educ. 2017 Feb 23;17(1):45.
97. Rao M. What's good for the climate is good for health: the role of doctors in addressing climate change. Natl Med J India. 2010 Feb;23(1):1–3.
98. Rice MB. Climate change at the bedside? Observations from an ATS membership survey. Ann Am Thorac Soc. 2015 Feb;12(2):245–6.
99. Ring W. Inspire Hope, Not Fear: Communicating Effectively About Climate Change and

Health. Ann Glob Health. 2015 Jun;81(3):410–5.
100. Roberts I, Stott R, Climate and Health Council executive. Doctors and climate change. BMJ. 2010 Nov 17;341:c6357.
101. Rogers W. Moral responsibility in medicine: where are the boundaries? Lancet. 2020 Aug 8;396(10248):373–4.
102. Rosenblatt RA. Ecological change and the future of the human species: can physicians make a difference? Ann Fam Med. 2005 Apr;3(2):173–6.
103. Sarfaty M, Abouzaid S. The physician's response to climate change. Fam Med. 2009 May;41(5):358–63.
104. Sarfaty M, Bloodhart B, Ewart G, Thurston GD, Balmes JR, Guidotti TL, et al. American Thoracic Society member survey on climate change and health. Ann Am Thorac Soc. 2015 Feb;12(2):274–8.
105. Sarfaty M, Kreslake J, Ewart G, Guidotti TL, Thurston GD, Balmes JR, et al. Survey of International Members of the American Thoracic Society on Climate Change and Health. Ann Am Thorac Soc. 2016 Oct;13(10):1808–13.
106. Sayre L, Rhazi N, Carpenter H, Hughes NL. Climate change and human health: the role of nurses in confronting the issue. Nurs Adm Q. 2010 Dec;34(4):334–42.
107. Schachtel A, Boos MD. Pediatric dermatology and climate change: An argument for the pediatric subspecialist as public health advocate. Pediatr Dermatol. 2019 Jul;36(4):564–6.
108. Schwartz BS, Parker C, Glass TA, Hu H. Global environmental change: what can health care providers and the environmental health community do about it now? Environ Health Perspect. 2006 Dec;114(12):1807–12.
109. Schwerdtle P. Nurse initiated hospital greening. Aust Nurs Midwifery J. 2015 Aug;23(2):32.
110. Sendall MC, Lidstone J, Fleming M, Domocol M. Nurses and teachers: partnerships for green health promotion. J Sch Health. 2013 Jul;83(7):508–13.
111. Sheffield PE, Durante KT, Rahona E, Zarcadoolas C. Emerging roles of health care providers to mitigate climate change impacts: a perspective from East Harlem, New York. Health Hum Rights. 2014 Jun 14;16(1):113–21.
112. Shelton CL, McBain SC, Mortimer F, White SM. A new role for anaesthetists in environmentally-sustainable healthcare. Anaesthesia. 2019 Sep;74(9):1091–4.
113. Shumer D. Doctor as environmental steward. Wilderness Environ Med. 2009;20(1):91.
114. Sibbald B. Physicians' roles on the front line of climate change. CMAJ. 2013 Feb 19;185(3):195.
115. Singleton JA, Lau ETL, Nissen LM. Waiter, there is a drug in my soup - using Leximancer® to explore antecedents to pro-environmental behaviours in the hospital pharmacy workplace. Int J Pharm Pract. 2018 Aug;26(4):341–50.
116. Solomon CG, LaRocque RC. Climate Change - A Health Emergency. N Engl J Med. 2019 Jan 17;380(3):209–11.
117. Storz MA, Heymann EP. USA withdrawal from Paris climate agreement: requiring unity among physicians worldwide more than ever before? Intern Med J. 2017 Oct;47(10):1218–9.
118. Storz MA. Mitigating climate change: using the physician's tool of the trade. Br J Gen Pract. 2019 Nov;69(688):557.
119. Sullivan-Marx E, McCauley L. Climate Change, Global Health, and Nursing Scholarship. J

Nurs Scholarsh. 2017 Nov;49(6):593–5.
120. Swartz MK. Taking on Climate Change Through a Health Care Lens. J Pediatr Health Care. 2019 Dec;33(6):623.
121. Teherani A, Nishimura H, Apatira L, Newman T, Ryan S. Identification of core objectives for teaching sustainable healthcare education. Med Educ Online. 2017;22(1):1386042.
122. Thompson M. Health implications of climate change. Oreg Nurse. 2004 Sep;69(3):13.
123. Tran V, Humphrey K, Cobbett J. Climate corrosion: Heroes wanted - Enquire within. Emerg Med Australas. 2019 Apr;31(2):271–3.
124. Ulhøi JP, Ulhøi BP. Beyond climate focus and disciplinary myopia. The roles and responsibilities of hospitals and healthcare professionals. Int J Environ Res Public Health. 2009 Mar;6(3):1204–14.
125. Valentine-Maher SK, Butterfield PG, Laustsen G. Environmental Health: Advancing Emancipatory Policies for the Common Good. ANS Adv Nurs Sci. 2018 Mar;41(1):57–69.
126. Valois P, Blouin P, Ouellet C, Renaud J-S, Bélanger D, Gosselin P. The Health Impacts of Climate Change: A Continuing Medical Education Needs Assessment Framework. J Contin Educ Health Prof. 2016;36(3):218–25.
127. Voelker R. Climate change puts children in jeopardy. JAMA. 2009 Jun 3;301(21):2197–9.
128. Vogel L. Global physician groups call on profession to address environmental health threats. CMAJ. 2019 Mar 25;191(12):E346.
129. Völker M, Hunchangsith P. Drivers of Physicians’ Engagement in Addressing Eco-health Problems. Ecohealth. 2018 Dec;15(4):853–63.
130. Walden J, Wenzel L. Beyond Climate Change: Why Medical Institutions Should Divest From Fossil Fuels. N C Med J. 2016 Apr;77(2):146.
131. Wasco JJ. Strategies for Teaching Online RN-to-BSN Students the Health Impacts of Climate Change. Creat Nurs. 2019 Aug 15;25(3):e1–8.
132. Wellbery CE. Climate Change Health Impacts: A Role for the Family Physician. Am Fam Physician. 2019 Nov 15;100(10):602–3.
133. Wellbery C, Sheffield P, Timmireddy K, Sarfaty M, Teherani A, Fallar R. It’s Time for Medical Schools to Introduce Climate Change Into Their Curricula. Acad Med. 2018 Dec;93(12):1774–7.
134. While A. Climate change should matter to nurses. Br J Community Nurs. 2006 Oct;11(10):454.
135. Wilson L, Black D, Veitch C. Heatwaves and the elderly - The role of the GP in reducing morbidity. Aust Fam Physician. 2011 Aug;40(8):637–40.
136. Xie E, de Barros EF, Abelsohn A, Stein AT, Haines A. Challenges and opportunities in planetary health for primary care providers. Lancet Planet Health. 2018 May;2(5):e185–7.
137. Emerging Leaders Tackling the Challenges of Environmental Health. Creat Nurs. 2019 Nov 1;25(4):270–4.
